# Supplementary material for: Functional in vivo characterization of sox10 enhancers in neural crest and melanoma development
Source: Commun Biol. 2021 Jun 7;4:695. doi: 10.1038/s42003-021-02211-0 (PMC8184803; doi:10.1038/s42003-021-02211-0)
Supplement: Supplementary file 2 — Supplementary Information [file 42003_2021_2211_MOESM2_ESM.pdf]

Supplemental Figures

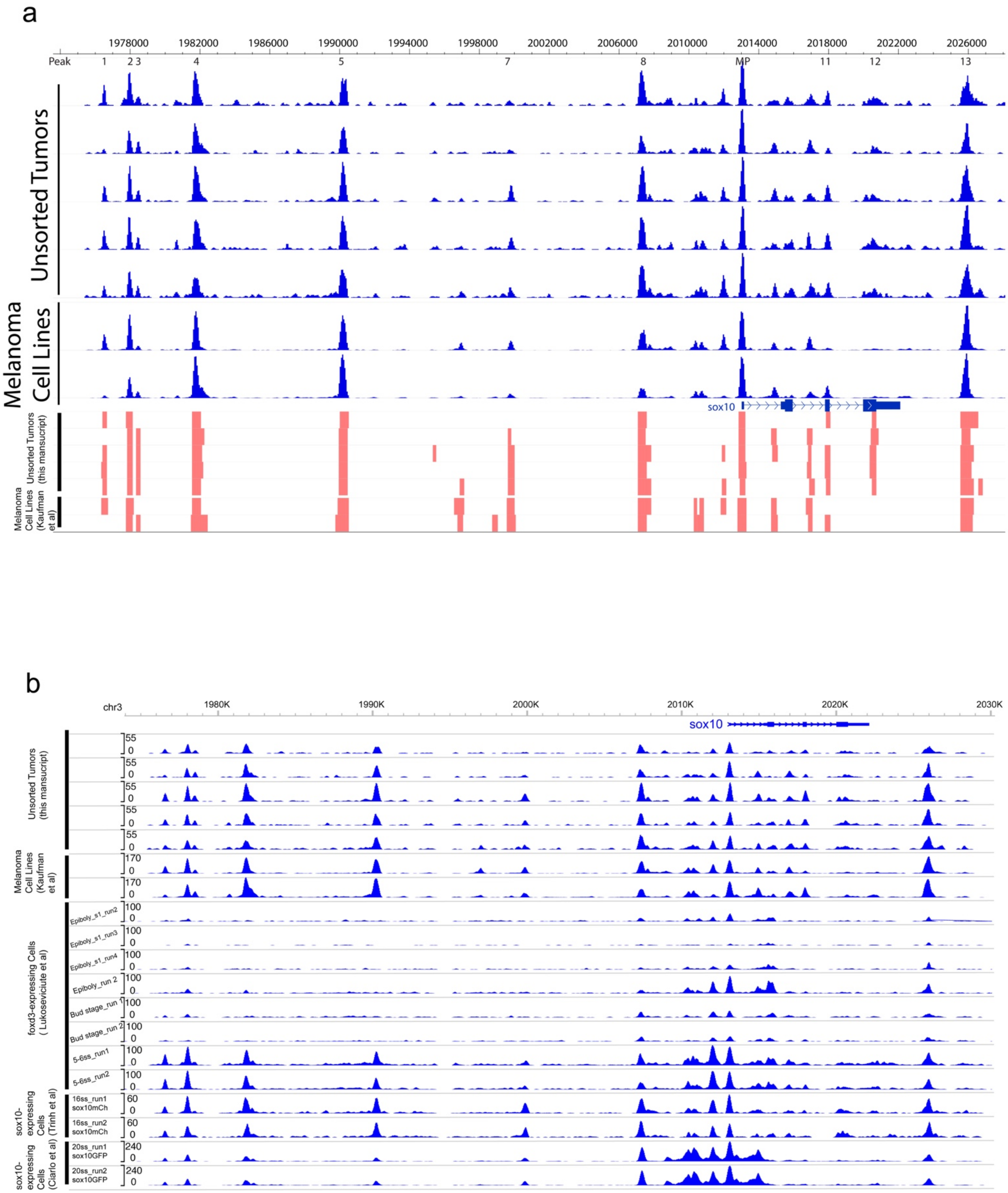

Supplemental Figure 1: ATAC-Seq peaks are consistent across tumors A) ATAC-Seq peaks surrounding the zebrafish *sox10* locus. Zebrafish melanoma cell line ATAC-Seq data aligns with tumor sample data. Red bars indicate in the lower portion of the figure show locations of significant peaks called using MACS2. B) Comparison of melanoma and neural crest ATAC-Seq. Putative transcriptional regulatory regions/accessible domains near sox10 from in vivo isolated zebrafish melanoma tumors (this report) and melanoma cell lines (Kaufman et al, Science, 2016) are also present in embryonic neural crest by 5-6 somite stage but not earlier at

bud and 75% epiboly in foxd3-expressing cells (from Lukoseviciute et al, Dev Cell, 2018). These regions also remain open in later sox10-positive cells at 16 and 20 somite stages (from Trinh et al, Cell Reports, 2017 and Ciarlo et al, eLife, 2017, respectively)

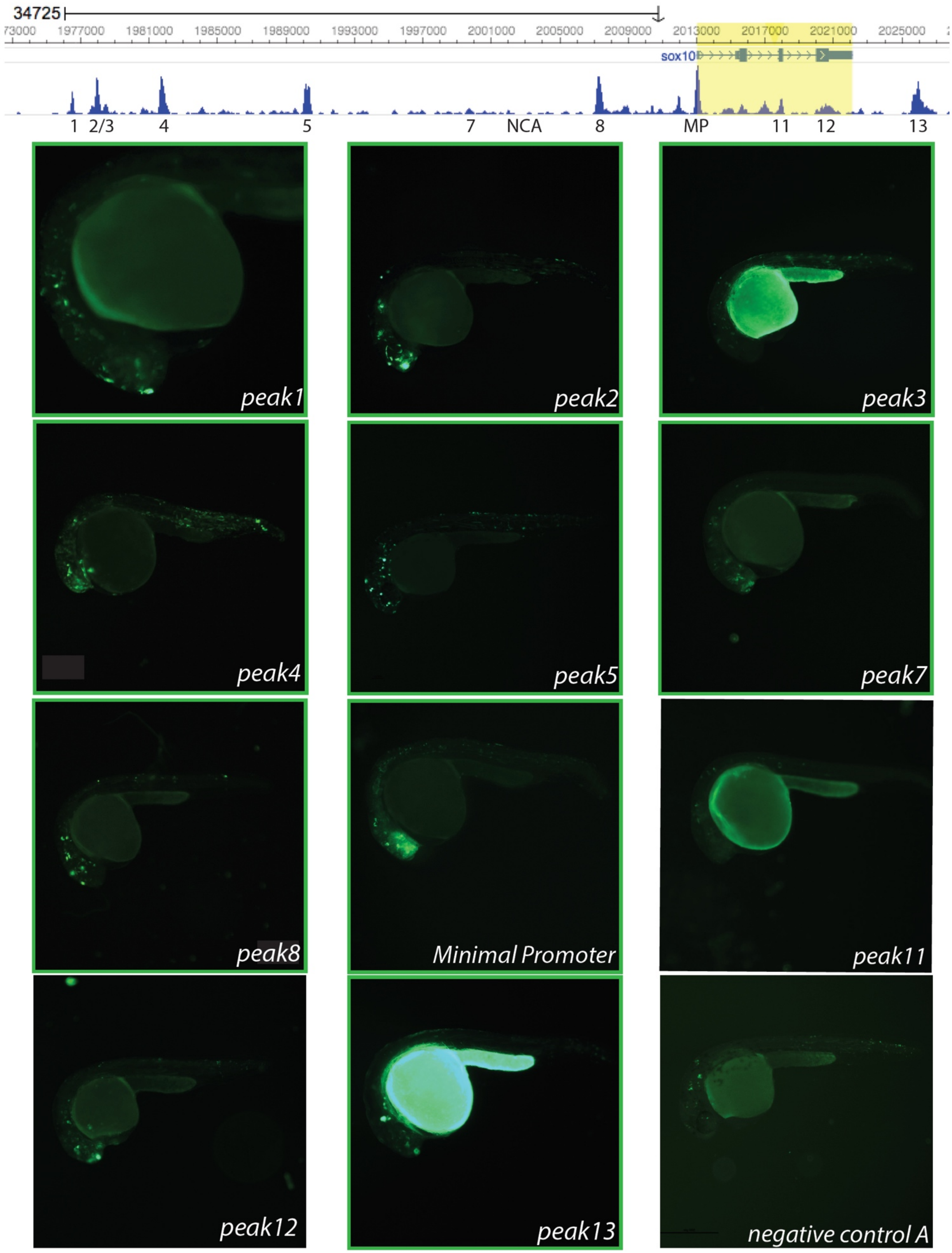

Supplemental Figure 2: 9 out of 11 peaks are active embryonically A) Regions of open chromatin within the *sox10* coding and non-coding genomic locus. NCA = negative control, MP = minimal promoter. **B)** 1 dpf images of F0 mosaic embryos expressing putative *sox10* enhancer assay reporters. Images lined in green indicate constructs that show some level of embryonic activity.

```
Enhancer5_sox10.ape from 735 to 1529
Alignment to
peak5_cloned.ape--  Matches:661; Mismatches:8; Gaps:126; Unattempted:0

              *         *         *         *         *         *         *         *         *         *
    735>agtcaatctgacagggtattgttgagtttttggaaatattcaaagcattttcccaaaatatgtgtcaaaacaagatttgtcaccaaaaatcatcctattt>834
      1>agtcaatctgacagggtattgttga gtttttggaaatattcaaagcattttcccaaaatatgtgtcaaaacaagatttgtcaccaaaaatcatcctattt>100

              *         *         *         *         *         *         *         *         *         *
    835>gtggtcaaaataactcttaaaattccccagagctgatgaaaacgacccaacagcacacacacgctacagtgttttcctctggacagactgactgggtgat>934
    101>gtggtcaaaataactcttaaaattccccagagctgatgaaaacgacccaacagcacacacacgctacagtgttttcctctggacagactgactgggcgat>200

              *         *         *         *         *         *         *         *         *         *
    935>GTGAAAGCTCTCTGCACCTGTATCCATGTGTGGTGGGCTCCTGATCATTCATTTATGCTCAGCGCTGTGAGAAACGGGCCTGAGAATCAGATGCTCTGCT>1034
    201>gtgaaagctctctgcacctgtatccatgtgtggtgggctcctgatcattcatatatgctcagcgctgtgagaaacgggcctgagaATCAGATGCTCTGCT>300

              *         *         *         *         *         *         *         *         *         *
   1035>CTCTGGATCAGCGTGACCTCAGAAATGAAGACGAGAGTCACAAGGCCGCGGCCGCCCGAGGGCAGAGACGTTTACGAGAGCGCAGAGACACACAGAAAGA>1134
    301>CTCTGgatcagcgtgacctcagaaatgaagacgagagtcacaaggccgcggc cgcccgagggcagagacgtttacgagagcgcagagacacacagaaaga>400

              *         *         *         *         *         *         *         *         *         *
   1135>GCCTTTTGTCGTCTGAATTCTTCCGCATCATTTTCTGCATTTCATCAGCTGTGAGTCAAACTCACCCACAAATCTCATTCCTGCCCGCTCTGTGTTTATC>1234
    401>gccttttgtcgtctgaattcttccgcatcattttctgcatttcatcagctgtgagtcaaactcacccacaaatctcattcctgcccgctctgtgtttatc>500

              *         *         *         *         *         *         *         *         *         *
   1235>ACATCACTCACACACACACACAACTGCTCAAATACTGATAAAAACACACACACACACACACACTCATAAATACACACACAAGAACTCAAAATGCTCA>1334
    501>acatcactcac--acacacacaactgctcaaatactgataaaaacacacacacacac--------tcataaatacacacaca------------------>572

              *         *         *         *         *         *         *         *         *         *
   1335>AACACTGATAAACACGCACACACATTCTTAAACACACATACTGATAAACACACactcataaacatgcacaaacacacacaaacactgataaacacacata>1434
    573>------------cacacacacaca----aaaacagaca-actg-----------ct----------------------caaatactg------------->609

              *         *         *         *         *         *         *         *         *
   1435>ttctcataaacacacacacaagcacacacactttctcgttaacatttaggaacaaacactgatacacactgacacacaagtaatcaaaaccgcac>1529
    610>-----ataaacacacacacacacacacacact----------cat-----aa---------atac------acacacaagtaatcaaaaccgcac>669
```

Supplemental Figure 3: ***peak5*** **cloned nucleotide sequence**

Zebrafish wild-type reference sequence (795 bp – top line) aligned to cloned *peak5* sequence (669 bp – bottom line). Capitalized nucleotide sequence in top reference sequence indicated region annotated as a peak by ATAC-Seq.

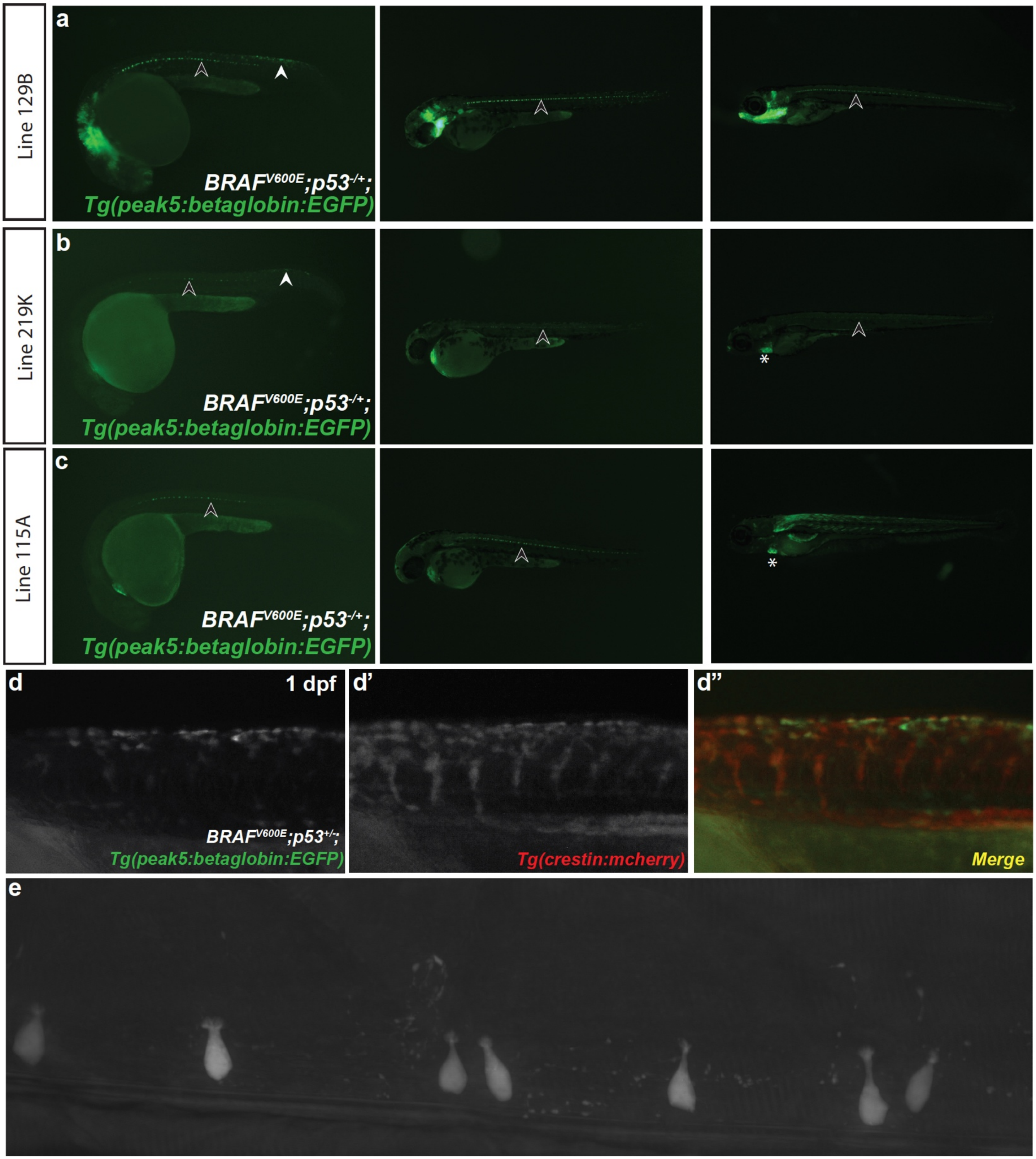

Supplemental Figure 4: ***peak5*** **labels a subset of NCCs in multiple stable transgenic lines and a subset of ventral Kolmer-Agduhr neurons in the spinal cord**

a) In Line 129B, NCCs, KA neurons, and cells in the cranial CNS are strongly labeled with EGFP at 1 dpf. Labeling of some NCCs is maintained at 2 dpf, and by 5 dpf EGFP is primarily localized within KA neurons and parts of the head. **b)** Line 219K exhibits EGFP localization in some premigratory NCCs, KA neurons, and the heart at 1 dpf. At 5 dpf, EGFP is mainly localized in KA neurons and the heart. **c)** Line 115A displays signs of ectopic *EGFP* expression. At 1 dpf, EGFP localization is mainly visible in KA neurons and the heart. By 2

dpf, EGFP is also localized in the muscles, which is more prominent at 5 dpf. KA neurons and the heart are also labeled at 2 dpf and 5 dpf. **d)** *peak5* (Stable Line A) is active in cells with NCC morphology and pre-migratory NCC localization at 1 dpf. **d'-d'')** These EGFP positive cells co-label with *Tg(crestin:mCh)* positive cells. **e)** Maximum projection confocal image of *peak5:betaglobin:EGFP* labeled KA neurons in a stable transgenic line.

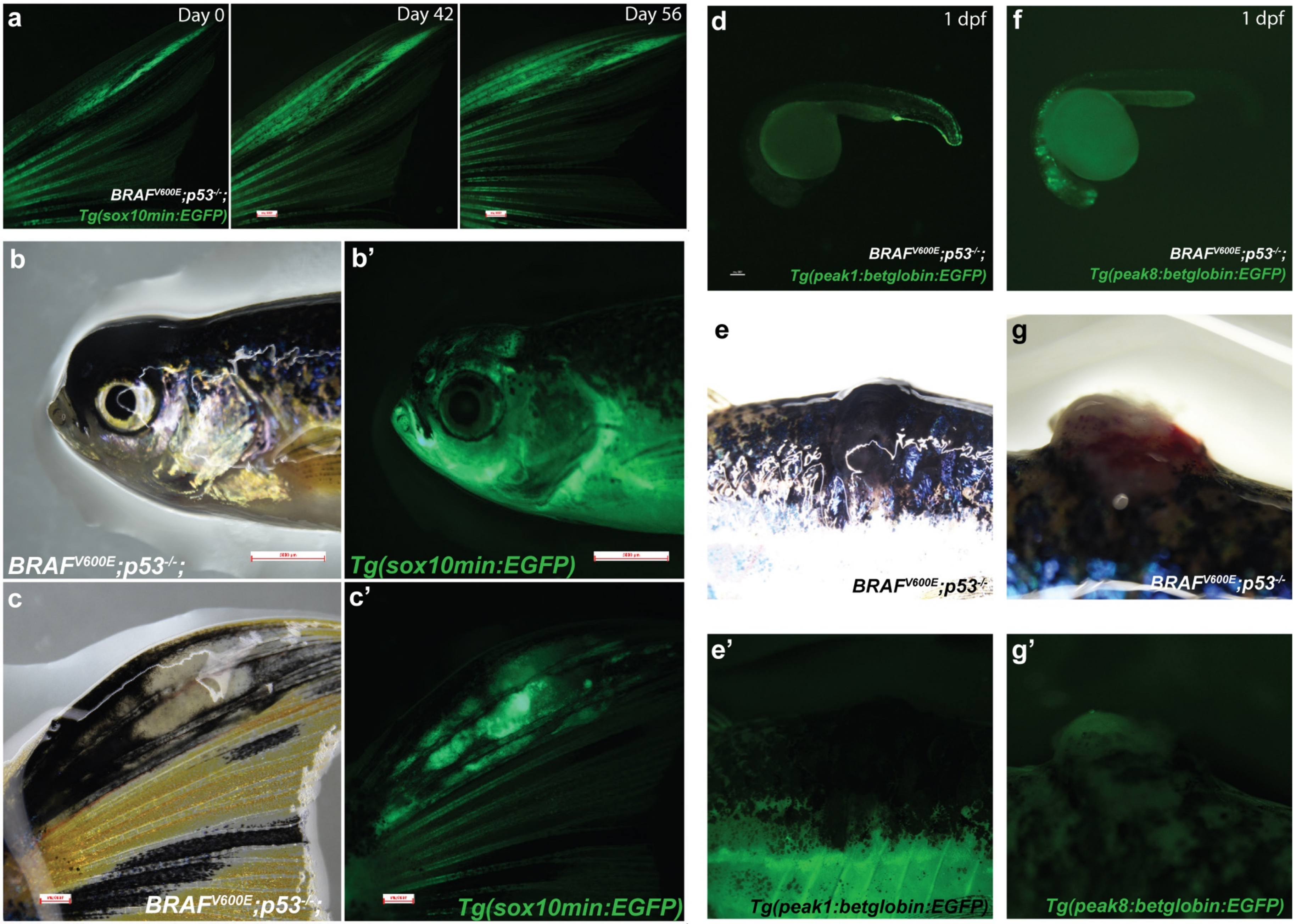

Supplemental Figure 5: The *sox10* minimal promoter is active in melanoma. *peak1* and *peak8* are not active in melanoma. A) The *sox10* minimal promoter (MP) is active in melanoma precursor lesions. **B-B')** The *sox10* minimal promoter is active in melanoma tumors on the head and

C-C') on the tail. **D)** One *peak1* stable line is active embryonically in the fin mesenchyme. **E-E')** *peak1* is not active in melanoma tumors. **F)** A representative *peak8* stable line is active in the CNS embryonically. **G-G')** *peak8* is not active in nearly all melanoma tumors.

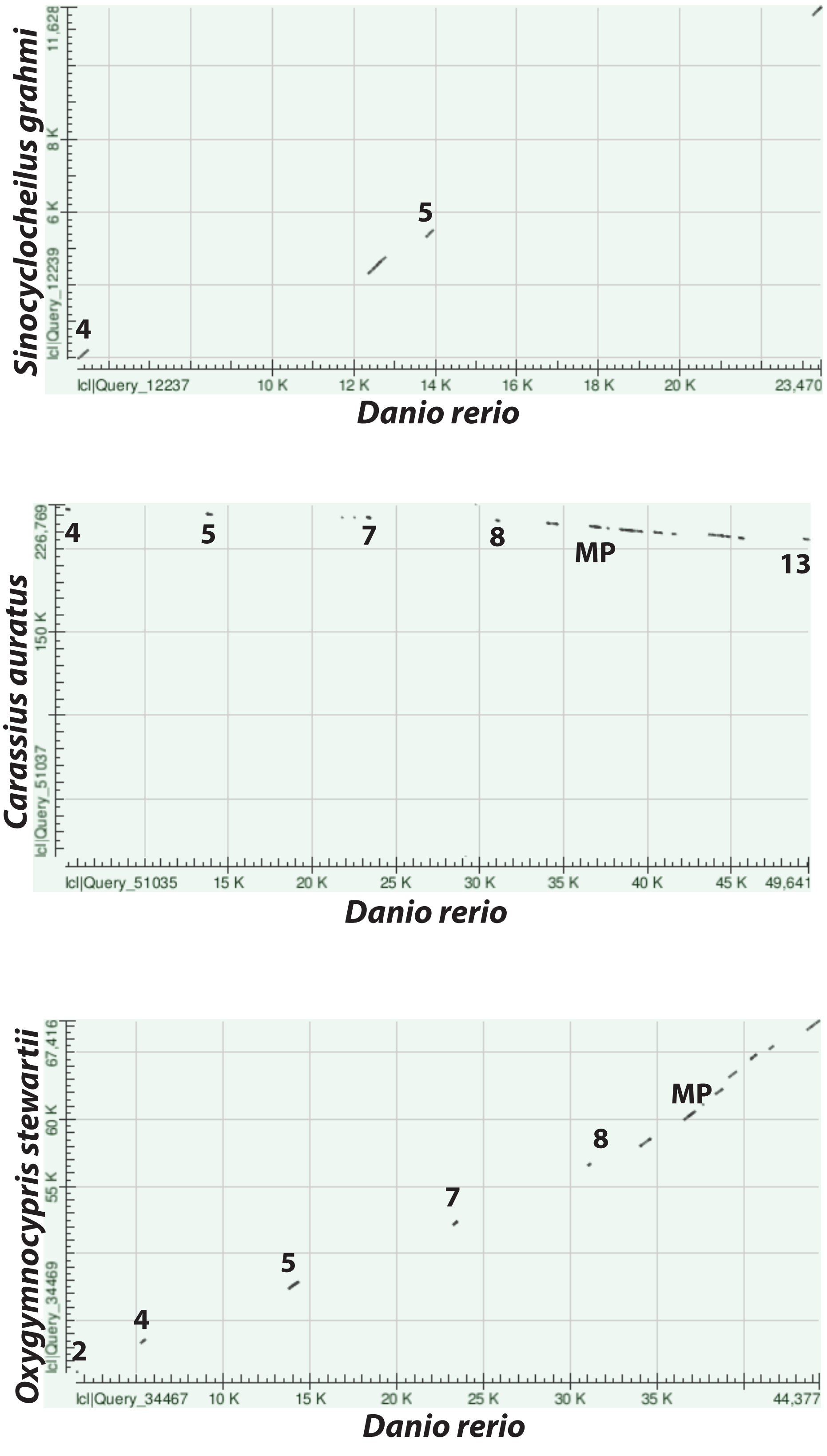

Supplemental Figure 6: Conservation of *sox10* enhancers across members of the *Cyprinidae* family. Dot-matrix view of the alignments of scaffolds containing *peak5* conservation compared to the same *sox10* genomic locus in zebrafish. Numbers indicate regions where peak sequences are conserved. MP = minimal promoter.

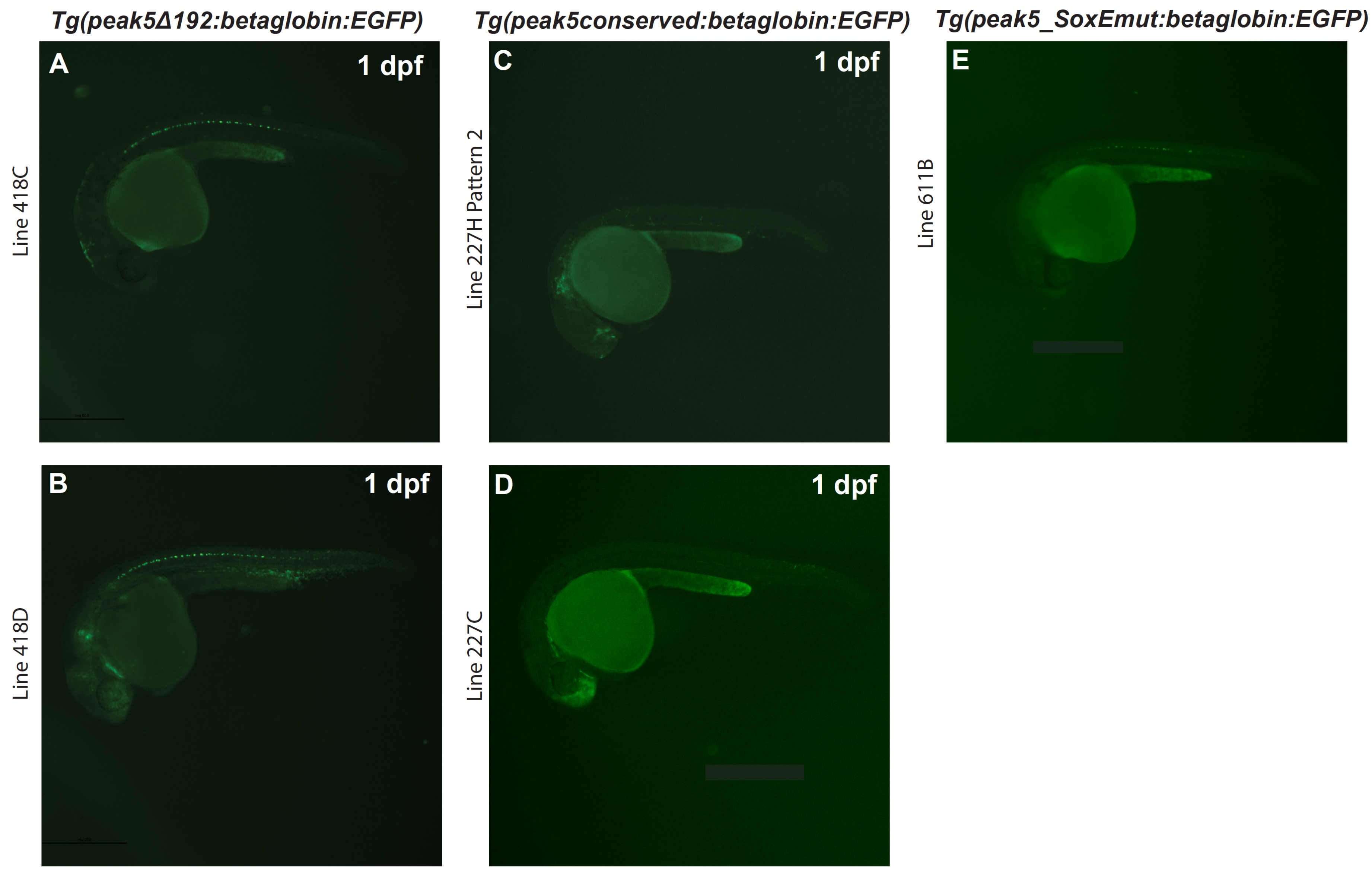

Supplemental Figure 7: Additional *Tg(peak_conserved:betaglobin:EGFP)* and *Tg(peak5Δ192:betaglobin:EGFP)* and *Tg(peak5_SoxEmut:betaglobin:EGFP)* stable lines. A and B) *Tg(peak5Δ192:betaglobin:EGFP)* stable lines exhibit robust *EGFP* expression in KA neurons at 1 dpf. **C)** A second pattern of expression identified from a single founder for *Tg(peak5_conserved:betaglobin:EGFP)* Line 227H. **D)** An additional *Tg(peak5_conserved:betaglobin:EGFP)* stable line exhibits EGFP localization in posterior dorsal NCCs, but does not exhibit any EGFP localization in KA neurons.

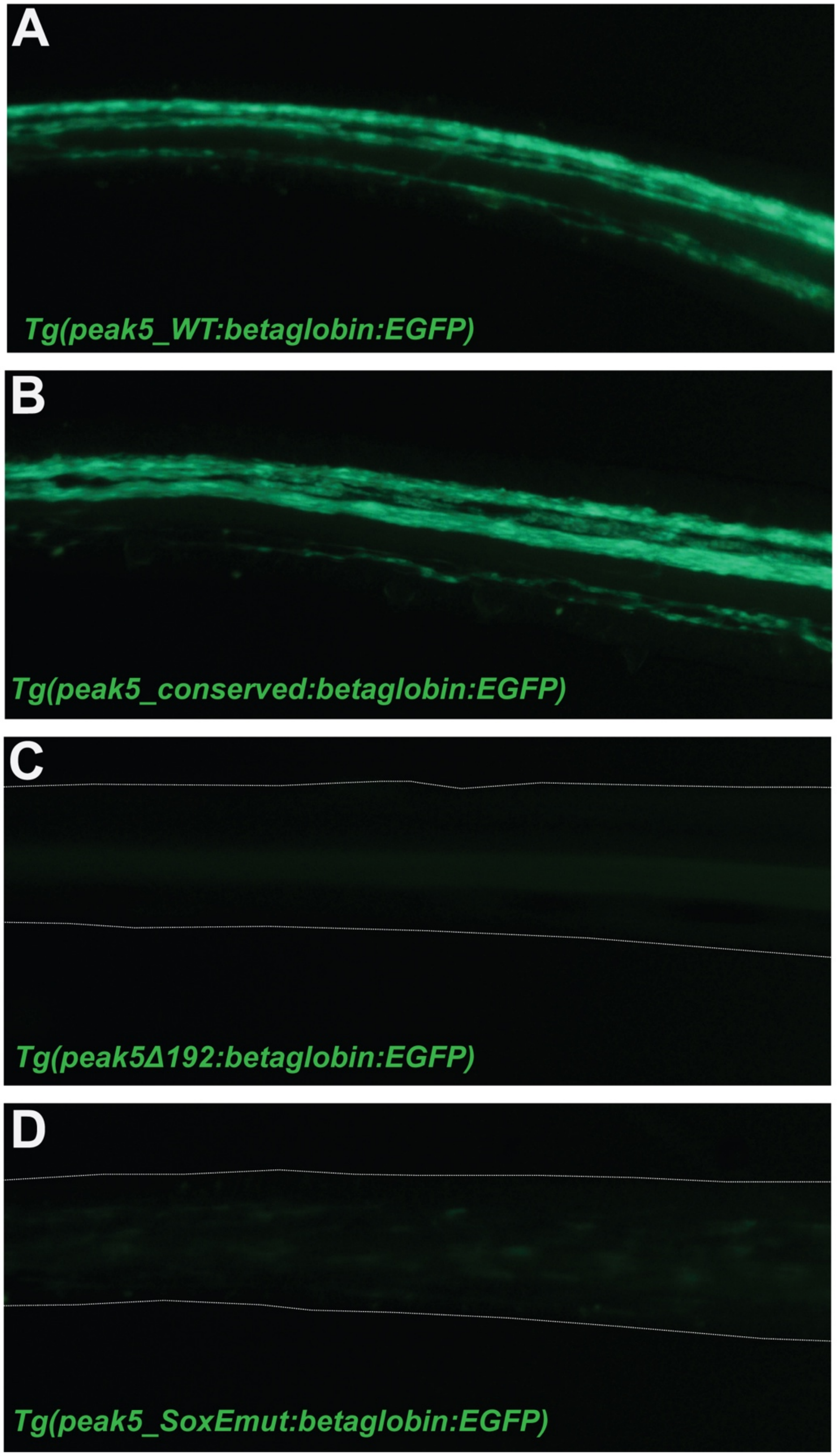

Supplemental Figure 8: Mutation of *peak5* affects *EGFP* expression in barbels in stable transgenic lines
A) Wild-type *peak5* exhibits robust activity in *sox10* expressing peripheral nerves in barbels. **B)** *Tg(peak5_conserved:betaglobin:EGFP)* stable lines exhibit similar *EGFP* expression in peripheral nerves in barbels as compared to wild-type *peak5*. **C)** *EGFP* expression is not detectable in *Tg(peak5Δ192:betaglobin:EGFP)* stable lines. **D)** *Tg(peak5_SoxEmut:betaglobin:EGFP)* lines do not exhibit EGFP expression in peripheral nerves. Some low level *EGFP* expression is present in these barbels, but not as robust compared to wild-type *peak5* labeled nerves in barbels and also does not appear to correspond to where nerves are present, as in *Tg(WTpeak5:betaglobin:EGFP)* and *Tg(peak5_conserved:betaglobin:EGFP)* lines.

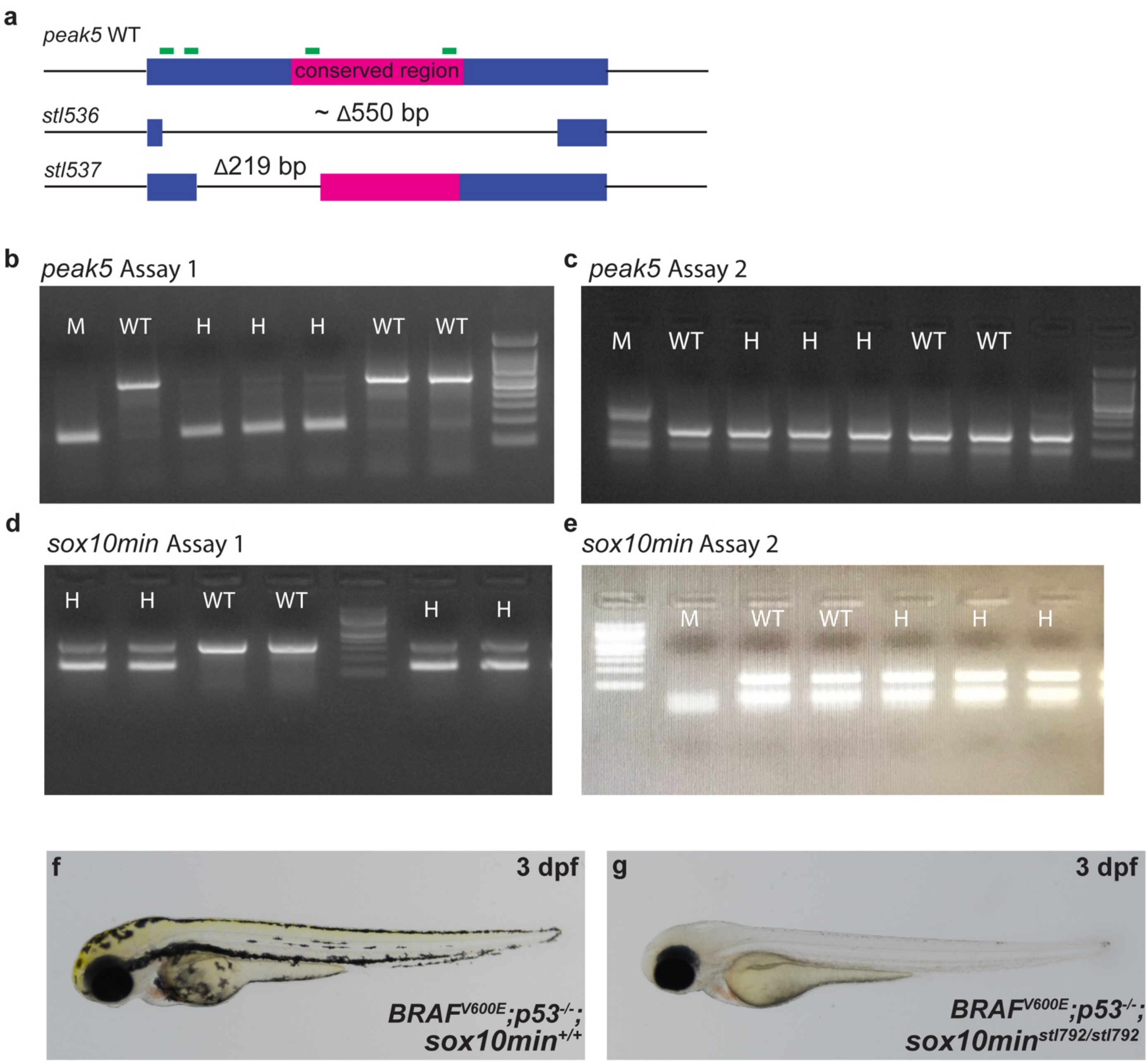

Supplemental Figure 9: *peak5* and *sox10min* deletion allele genotyping assays
a) Two additional identified *peak5* alleles. *stl536* has a ~550 bp deletion. *stl537* has a 219 bp deletion. **b)** Genotyping assay 1 for *peak5* allele *stl538*. *peak5* assay 1 primers amplify a 533 bp wild-type (WT) band or a 164 bp mutant band (M). Heterozygous fish (H) exhibit both WT and M bands. However, distinguishing homozygous mutants from heterozygous fish is not always clear. Therefore, **c)** *peak5* assay 2 primers amplify the conserved sequence, 192 bp, of *peak5* that is absent in the *stl538* allele. Subsequently, homozygous mutants do not have 192 bp band present in this assay, whereas wild-type and heterozygous fish do exhibit a band. **d)** Genotyping assay 1 for *sox10min* allele *stl792*. *sox10min* assay 1 primers amplify a 350 bp wild-type (WT) band or a 150 bp mutant band. Heterozygous fish (H) exhibit both WT and M bands. However, distinguishing homozygous mutants from heterozygous fish is not always clear. Therefore, **e)** *sox10min* assay 2 primers amplify a 136 bp sequence that is absent in the *stl792* allele. Subsequently, homozygous mutants (M) do not have 136 bp band present in this assay, whereas wild-type (WT) and heterozygous (H) fish do exhibit a band. **f)** At 3 dpf, melanocytes are present in wild-type embryos. **g)** Melanocytes are not present in 3 dpf *sox10min*$^{stl792/stl792}$ homozygous mutants

Supplementary Table 1: Numbers of fish screened, and number of EGFP positive and EGFP negative tumors for each stable line examined in this study

Stable Line	Number of Fish Screened	Number of EGFP+ Tumors	Number of EGFP- Tumors
peak5 LineA	20	22	2
peak5 LineB	14	14	1
peak5 LineC	17	19	1
peak5 Line129B	10	12	0
***peak5* Totals**	**61**	**67**	**4**
peak5Δ192 Line328C	9	0	10
peak5Δ192 Line418C	9	0	10
peak5Δ192 Line418D	14	3	13
***peak5Δ192* Totals**	**32**	**3**	**33**
peak5_SoxEmut Line64J	**6**	**2**	**4**
peak5_SoxEmut Line611B	**5**	**0**	**7**
***peak5_SoxEmut* Totals**	**11**	**2**	**11**
sox10min LineA1	5	5	0
sox10min LineC	16	15	4
sox10min Line103B	6	5	2
sox10min Line912E	7	6	1
sox10min Line1228AC	7	6	1
sox10min Line1228A	1	1	0
***sox10min* Totals**	**42**	**38**	**8**
peak1 LineA	6	0	6
***peak1* Totals**	**6**	**0**	**6**
peak8 Line918E	10	0	11
peak8 Line918I	9	0	9
peak8 Line918F	6	1	5
***peak8* Totals**	**25**	**1**	**25**

Supplementary Table 2: ATAC-Seq peak locations in zebrafish (danRer10)

Transcription start site (TSS) = Chr3: 2013057. The distance of each amplified peak from the sox10 TSS was calculated from the middle of each peak.

Peak Name	Annotated Location	Amplified Location	Distance to TSS (Amplified sequence)
peak 1	Chr3: 1976452-1976658	Chr3: 1976387-1976752	-36.4 KB
peak 2	Chr3: 1977838-1978112	Chr3: 1977785-1978218	-35 KB
peak 3	Chr3: 1978414-1978643	Chr3: 1978343-1978649	34.5 KB
peak 4	Chr3: 1981603-1982037	Chr3: 1981487-1982249	-31.1 KB
peak 5	Chr3: 1990057-1990509	Chr3: 1989857-1990651	-22.8 KB
peak 7	Chr3: 1999674-2000029	Chr3: 1999609-2000086	-13.2 KB
peak 8	Chr3: 2007199-2007580	Chr3: 2006988-2007878	-5.6 KB
peak 11	--	Chr3: 2017817-2018186	4.9 KB
peak 12	--	Chr3: 2020003-2020873	7.3 KB
peak 13	Chr3: 2025633-2026297	Chr3: 2025361-2026723	12.9 KB
minimal promoter	Chr3: 2012890-2013261	Chr3: 2012920-2013269	37 BP
NEG CONTROL A	--	Chr3: 2002002-2002703	-10.7 KB
NEG CONTROL B	--	Chr3: 1936346-1937052	-76.3 KB

Supplementary Table 3: List of cloning primers used in this study

Peak Name		Sequence (5'-3')	Notes
peak 1			376 bp amplified
	F	acagtgggaaatgaacagcag	
	R	gaagagctgcgttctgctttg	
peak 2			434 bp amplified
	F	cattacttcgcaggttttgtctg	
	R	agtgaattttcatttagtgggctg	
peak 3			311 bp amplified
	F	acaggtttacagaggttaacag	
	R	tggaatataaacagaacaaagtcc	
peak 4			743 bp amplified
	F	tagaaacacaacagaagtgctg	
	R	aagccagaatacagcatagc	
peak 5			669 bp amplified
	F	agtcaatctgacagggtattg	
	R	gtgcggttttgattacttgtg	
peak 7			478 bp amplified
	F	caacaacaatctcagagtgatctc	
	R	ttgacttctactgtgtgtgaac	
peak 8			~915 bp amplified
	F	acatactgtgaacatttcctgaatc	
	R	tgtgccatcaattgtgaatcag	
peak 11			382 bp amplified
	F	cacatgtttcagactgctgaac	
	R	ttgatcagttaaatgtgtgtttgag	
peak 12			871 bp amplified
	F	tgctcaggtcagagtcacagc	
	R	tcagtttgtgtcgattgtggtgc	
peak 13			
	F	acttgttaacatgcagctgtca	

R	gttgcgtgagtgtgtacatatg	
sox10 Minimal Promoter		351 bp amplified
F	**acaaaaaagcaggctcgcta** cctgtgagaggccaaatattac	bold = Gibson Assembly overlap
R	**cttgctcaccatggtggcga** agtttctccgctagacagtg	bold = Gibson Assembly overlap
**Negative Control A**		
F	gtacacctgtaatcaaagctgc	
R	gacactgtaatgtacagatgttgc	
**Negative Control B**		702 bp amplified
F	tccactgtaagaagcgtaatagg	
R	gaagaagcatagagctgaaagc	
***peak5* Conserved Sequence**		192 bp amplified
F	cagcgtgacctcagaaatgaag	
R	gataaacacagagcgggcag	
**Peak 5 Plasmid Mutagenesis**	**Sequence (5'-3')**	**Notes**
**Conserved Deletion**		
F	ACATCACTCACACACACAC	
R	ATCCAGAGAGCAGAGCATC	
**SoxE Mutations**		
F	cct**gcgac**CGTCTGAATTCTTCCGCATC	Bold = SoxE mutations
R	ctc**gccac**GTGTGTCTCTGCGCTCTC	Bold = SoxE mutations

Supplementary Table 4: List of plasmids used and/or generated in this study

Plasmid Name	Purpose	Source
**5' Entry Clones**		
p5E*peak1*	Gateway Cloning	Kaufman Lab
p5E*peak2*	Gateway Cloning	Kaufman Lab
p5E*peak3*	Gateway Cloning	Kaufman Lab
p5E*peak4*	Gateway Cloning	Kaufman Lab
p5E*peak5*	Gateway Cloning	Kaufman Lab
p5E*peak7*	Gateway Cloning	Kaufman Lab
p5E*peak8*	Gateway Cloning	Kaufman Lab
p5E*peak11*	Gateway Cloning	Kaufman Lab
p5E*peak12*	Gateway Cloning	Kaufman Lab
p5E*peak13*	Gateway Cloning	Kaufman Lab
p5EnegativecontrolA	Gateway Cloning	Kaufman Lab
p5EnegativecontrolB	Gateway Cloning	Kaufman Lab
p5E*peak5_conserved*	Gateway Cloning	Kaufman Lab
p5E-MCS	Gateway Cloning	Tol2 Kit
**Middle Entry Clones**		
pME*betaglobin:EGFP*	Gateway Cloning	Tamplin *et al.,* 2011
pENTR-EGFP2	Gateway Cloning	Addgene 22450
pMEsox10min	Gateway Cloning	Kaufman Lab
**3' Entry Clones**		
p3E-polyA	Gateway Cloning	Tol2 Kit
**Destination Vectors**		
pDestTol2pA2	Gateway Cloning	Tol2 Kit
**Enhancer Assay Plasmids**		
peak1:betaglobin:EGFP	Injection	Kaufman Lab
peak2:betaglobin:EGFP	Injection	Kaufman Lab
peak3:betaglobin:EGFP	Injection	Kaufman Lab
peak4:betaglobin:EGFP	Injection	Kaufman Lab
peak5:betaglobin:EGFP	Injection	Kaufman Lab
peak7:betaglobin:EGFP	Injection	Kaufman Lab
peak8:betaglobin:EGFP	Injection	Kaufman Lab
peak11:betaglobin:EGFP	Injection	Kaufman Lab
peak12:betaglobin:EGFP	Injection	Kaufman Lab
peak13:betaglobin:EGFP	Injection	Kaufman Lab
neg_controlA:betaglobin:EGFP	Injection	Kaufman Lab
neg_controlB:betaglobin:EGFP	Injection	Kaufman Lab
sox10min:EGFP	Injection	Kaufman Lab
peak5Δ192:betaglobin:EGFP	Injection	Kaufman Lab
peak5_conserved:betaglobin:EGFP	Injection	Kaufman Lab
peak5_SoxEmut:betaglobin:EGFP	Injection	Kaufman Lab

Supplementary Table 5: peak5 conservation coordinates across members of the Cyprinidae family

Species	Region of Conservation	Region Used for M-Coffee Alignment
Cyrprinus carpio	scaffold: LG6, chromosome 6:16144792-16145144	scaffold: LG6, chromosome 6:16144792-16145144
Sinocyclocheilus grahmi	scaffold888.1_15: 5319-5517	scaffold888.1_15: 5319-5517
Pimephales promelas	scaffold-281591: 449-637	scaffold-281591: 449-637
Carassius auratus	tig00035131: 220509-220798	tig00035131: 220509-220757
Oxygymnocypris stewartii	isolate Jianluoli-Novo-2018 ctg5171: 47377-47782	isolate Jianluoli-Novo-2018 ctg5171: 47377 - 47571

Supplementary Table 6: sgRNAs sequences for *peak5* and *sox10min* CRISPRs

	**Sox10 Minimal Promoter**	**Promoter**
gRNA1 (5')	taatacgactcactata **G**GCCCAGAGGGCGGTGATTTgttttagagctagaaatagc	T7
gRNA2 (5')	taatacgactcactataG **G**CGAAATCACCGCCCTCTGgttttagagctagaaatag	T7
gRNA3 (3')	taatacgactcactataG **G**CGGACTCTCGCGCTGGGCgttttagagctagaaatag	T7
	**Peak5**	
gRNA1 (5')	atttaggtgacactataGAGTTATTTTGACCACAAATgttttagagctagaaatagc	Sp6
gRNA2 (5')	taatacgactcactataGGTCGTTTTCATCAGCTCTGgttttagagctagaaatagc	T7
gRNA3 (3')	taatacgactcactata **G**GGGCAGGAATGAGATTTGTgttttagagctagaaatagc	T7
gRNA7 (5')	atttaggtgacactataGACGAGAGTCACAAGGCCGgttttagagctagaaatagc	SP6

Bold: mutated nucleotides from reference

5' lower case: promoter sequence

UPPERCASE: gRNA sequence

3' lowercase: tracr RNA sequence

Supplementary Table 7: List of genotyping primers

Note that the peak5 assay 1 primers are not able to amplify the stl536 allele.

Primer	Sequence (5'-3')	Notes	
**CRISPR Screening primers**			
peak5	tctgacagggtattgttgag	Amplifies 611 bp WT band	
	gcgtgtttatcagtgtttgagc		
peak5			
	tctgacagggtattgttgag	Amplifies 533 bp WT band	
	atcagtatttgagcagttgtg		
sox10min			
	cctgtgagaggccaaatattac	Amplifies 350 bp WT band	
	agtttctccgctagacagtg		
**Genotyping Primers**			
***peak5* Assay 1**			
F	tctgacagggtattgttgag	WT: 533 bp	**does not work for 319D
R	atcagtatttgagcagttgtg	Het: 164 bp and 533 bp	
		Mut: 164 bp	
***peak5* Assay 2**			
F	cagcgtgacctcagaaatgaag	WT and Het: 192 bp	
R	gataaacacagagcgggcag	Mut: No amplification at locus	
***sox10min* Assay 1**			
F	cctgtgagaggccaaatattac	WT: 350 bp	
R	agtttctccgctagacagtg	Het: 350 bp and 150 bp	
		Mut: 150 bp	
***sox10min* Assay 2**			
F	atcagagcgggacccgacagg	WT and Het: 136 bp	
R	ctcgcgctctgaatgcctgctc	Mut: No amplification at locus	
