# Supplementary material for: Functional in vivo characterization of sox10 enhancers in neural crest and melanoma development
Source: Commun Biol. 2021 Jun 7;4:695. doi: 10.1038/s42003-021-02211-0 (PMC8184803; doi:10.1038/s42003-021-02211-0)
Supplement: Supplementary file 3 — Description of Additional Supplementary Files [file 42003_2021_2211_MOESM3_ESM.pdf]

Description of Additional Supplementary Files

File name: Supplementary Data 1

Description: Numerical source data for quantification of EGFP reporter expression (Figure 4d, 5d), sox10 expression (Figure 6g, 6j), and stripe breaks per animal (Figure 6m, 6r).
