# Supplementary material for: Functional in vivo characterization of sox10 enhancers in neural crest and melanoma development
Source: Commun Biol. 2021 Jun 7;4:695. doi: 10.1038/s42003-021-02211-0 (PMC8184803; doi:10.1038/s42003-021-02211-0)
Supplement: Supplementary file 5 — Reporting Summary [file 42003_2021_2211_MOESM5_ESM.pdf]

nature research

Corresponding author(s): Charles K. Kaufman

Last updated by author(s): Apr 30, 2021

Reporting Summary

Nature Research wishes to improve the reproducibility of the work that we publish. This form provides structure for consistency and transparency in reporting. For further information on Nature Research policies, see our Editorial Policies and the Editorial Policy Checklist.

Statistics

For all statistical analyses, confirm that the following items are present in the figure legend, table legend, main text, or Methods section.

n/a	Confirmed	
☐	☒	The exact sample size (*n*) for each experimental group/condition, given as a discrete number and unit of measurement
☐	☒	A statement on whether measurements were taken from distinct samples or whether the same sample was measured repeatedly
☐	☒	The statistical test(s) used AND whether they are one- or two-sided<br>*Only common tests should be described solely by name; describe more complex techniques in the Methods section.*
☒	☐	A description of all covariates tested
☒	☐	A description of any assumptions or corrections, such as tests of normality and adjustment for multiple comparisons
☒	☐	A full description of the statistical parameters including central tendency (e.g. means) or other basic estimates (e.g. regression coefficient) AND variation (e.g. standard deviation) or associated estimates of uncertainty (e.g. confidence intervals)
☐	☒	For null hypothesis testing, the test statistic (e.g. *F*, *t*, *r*) with confidence intervals, effect sizes, degrees of freedom and *P* value noted<br>*Give P values as exact values whenever suitable.*
☒	☐	For Bayesian analysis, information on the choice of priors and Markov chain Monte Carlo settings
☒	☐	For hierarchical and complex designs, identification of the appropriate level for tests and full reporting of outcomes
☒	☐	Estimates of effect sizes (e.g. Cohen's *d*, Pearson's *r*), indicating how they were calculated

Our web collection on statistics for biologists contains articles on many of the points above.

Software and code

Policy information about availability of computer code

Data collection: NCBI BLAST with optimization for more dissimilar sequences with discontiguous megablast was used to compare the zebrafish peak5 sequence to selected members of the Cyprinidae family. TFBS predictions were identified with FIMO (http://meme-suite.org/tools/fimo) using the JASPAR core 2016 vertebrate list. SoxE dimeric binding sites were predicted with JASPAR (http://jaspar.genereg.net/search?q=&collection=CORE&tax_group=vertebrates), searching explicitly for Sox10 motifs using an 80% relative profile score threshold.

Data analysis: For ATAC-seq analysis: The reads were aligned to the GRCz10/danRer10 genome using BWA-MEM and sorted using SAMtools. Duplicate reads removed with Picard tools using the following parameters: ASSUME_SORTED=true, VALIDATION_STRINGENCY=LENIENT. The files were indexed with SAMtools then filtered for high quality alignments using the following parameters: -f 3, -F 4, -F 8, -F 256, -F 1024, -F 2048, -q 30. MACS2 was then used to identify peaks with the callpeak command with parameters -g 1.4e9, -q 0.05, --nomodel, --shift -100, --extsize 200.

For manuscripts utilizing custom algorithms or software that are central to the research but not yet described in published literature, software must be made available to editors and reviewers. We strongly encourage code deposition in a community repository (e.g. GitHub). See the Nature Research guidelines for submitting code & software for further information.

Data

Policy information about availability of data

All manuscripts must include a data availability statement. This statement should provide the following information, where applicable:

- Accession codes, unique identifiers, or web links for publicly available datasets
- A list of figures that have associated raw data
- A description of any restrictions on data availability

The data that support the findings of this study are available from the corresponding author upon request.

Field-specific reporting

Please select the one below that is the best fit for your research. If you are not sure, read the appropriate sections before making your selection.

☒ Life sciences ☐ Behavioural & social sciences ☐ Ecological, evolutionary & environmental sciences

For a reference copy of the document with all sections, see nature.com/documents/nr-reporting-summary-flat.pdf

Life sciences study design

All studies must disclose on these points even when the disclosure is negative.

Sample size	A sample size calculation was not performed. Sample size was determined based upon commonly accepted n numbers in the zebrafish developmental and melanoma fields.
Data exclusions	No data were excluded from analyses.
Replication	Three technical and biological replicates of injections were performed for each putative enhancer injection experiment. Two technical replicates and three technical replicates were performed for Negative Control A and Negative Control B, respectively. Multiple stable lines for peak5 (6 lines), peak5_conserved deletion (3 lines), peak5_conserved (2 lines), peak5_SoxEmut (2 lines), sox10_minimal promoter (6 lines), and peak8 (3 lines) were analyzed to assess if the transgenes reflected endogenous enhancer activity. For peak5 plasmid mutagenesis experiments, three technical and biological replicates were performed for the conserved deletion experiment and two technical and biological replicates were performed for the SoxE TFBS mutation experiment. For WISH experiments, one biological replicate and two technical replicates were performed for stl792 analysis and two biological replicates and five technical replicates were performed for stl538 analysis.
Randomization	Randomization was introduced through random mating of zebrafish containing our transgenes of interest to generate embryos for injection and transgene analyses, as well as adults for tumor analyses.
Blinding	The scorer was blinded to treatment groups when scoring peak5 plasmid mutagenesis experiments, WISH sox10 expression level, and stripe break numbers.

Reporting for specific materials, systems and methods

We require information from authors about some types of materials, experimental systems and methods used in many studies. Here, indicate whether each material, system or method listed is relevant to your study. If you are not sure if a list item applies to your research, read the appropriate section before selecting a response.

Materials & experimental systems

n/a	Involved in the study
☒	☐ Antibodies
☒	☐ Eukaryotic cell lines
☒	☐ Palaeontology and archaeology
☐	☒ Animals and other organisms
☒	☐ Human research participants
☒	☐ Clinical data
☒	☐ Dual use research of concern

Methods

n/a	Involved in the study
☒	☐ ChIP-seq
☒	☐ Flow cytometry
☒	☐ MRI-based neuroimaging

Animals and other organisms

Policy information about studies involving animals; ARRIVE guidelines recommended for reporting animal research

Laboratory animals	Zebrafish (Danio rerio). Adult zebrafish were crossed either as pairs or groups, and embryos were raised in egg water (5 mM NaCl, 0.17 mM KCl, 0.33 mM CaCl2, 0.33 mM MgSO4) at 28.5C. Larvae were staged at days post fertilization (dpf) and adults were staged at months post fertilization (mpf).
Wild animals	No wild animals were used in this study.
Field-collected samples	This study did not involve samples collected from the field.
Ethics oversight	Zebrafish were reared in accordance with Washington University IACUC animal protocols in the Washington University Zebrafish Consortium Facility.

Note that full information on the approval of the study protocol must also be provided in the manuscript.
